# Antibiotic prescribing patterns at outpatient clinics in Western and Coastal Kenya

**DOI:** 10.1371/journal.pgph.0004109

**Published:** 2025-01-03

**Authors:** Melanie Kiener, Caroline Ichura, Bryson A. Ndenga, Francis M. Mutuku, Christabel A. Winter, Victoria Okuta, Laura Mwambingu, Kevin Ogamba, Karren N. Shaita, Charles Ronga, Philip Chebii, Jael Amugongo, Said Malumbo, Omar Godana, Zainab Jembe, Charles Ng’ang’a, Mwangosho Mazera, A. Desiree LaBeaud

**Affiliations:** 1 Department of Medicine, Division of Infectious Diseases, Stanford University School of Medicine, Stanford, California, United States of America; 2 Department of Pediatrics, Division of Pediatric Infectious Diseases, Stanford University School of Medicine, Stanford, California, United States of America; 3 Kenya Medical Research Institute, Kisumu, Kenya; 4 Department of Environmental and Health Services, Technical University of Mombasa, Mombasa, Kenya; 5 Department of Pediatrics, Obama Children’s Hospital, Jaramogi Oginga Odinga Referral Hospital, Kisumu, Kenya; 6 Vector-Borne Diseases Unit, Msambweni County Referral Hospital, Msambweni, Kwale, Kenya; 7 Diani Health Center, Ukunda, Kwale, Kenya; Università degli Studi di Firenze: Universita degli Studi di Firenze, ITALY

## Abstract

Antimicrobial resistant pathogens are a leading cause of morbidity and mortality worldwide, with overuse and misuse of antimicrobials being key contributors. We aimed to identify factors associated with antibiotic prescriptions among patients presenting to clinics in Kenya. We performed a retrospective, descriptive cohort study of persons presenting to outpatient clinics in Western and Coastal Kenya, including symptoms, physical exams, clinician assessments, laboratory results and prescriptions. We reviewed 1,526 visits among 1,059 people who sought care from December 2019-February 2022. Median age was 16 (IQR 6–35) and 22% were under 5. 30% of malaria RDTs were positive and 3% of dengue RT-qPCRs were positive. Antibiotics were prescribed in 73% of encounters overall and in 84% among children under 5. In 48% of visits antibiotics were prescribed without a provisional bacterial diagnosis. In the multivariable model, factors associated with increased odds of an antibiotic prescription were the clinic in Western Kenya (OR 5.1, 95% CI 3.0–8.8), age less than or equal to 18 (OR 2.1, 95% CI 1.4–3.2), endorsement of cardiorespiratory symptoms (OR 5.2, 95% CI 3.2–8.3), a negative malaria RDT (OR 4.0, 95% CI 2.5–6.8), and a provisional diagnosis that could be bacterial in etiology (OR 5.9, 95% CI 3.5–10.3). High rates of antibiotic prescriptions are common even when associated diagnoses are not bacterial. Compared to our 2014–2017 cohort, we found higher rates of antibiotic prescriptions among children. Improved diagnostics to rule in alternative diagnoses as well as stewardship programs are needed.

## Introduction

Antimicrobial resistant (AMR) pathogens are a leading cause of morbidity and mortality globally, with the highest burden in sub-Saharan Africa [1,2]. The problem of AMR is multifaceted, but key contributors are the overuse and misuse of antibiotics in healthcare settings, where a global increase was seen most recently during the COVID-19 pandemic [3,4]. Approaching the problems of antibiotic overuse and misuse are particularly challenging in low- and middle-income countries (LMICs), where sepsis is a leading cause of childhood mortality, the differential diagnosis of acute febrile illness (AFI) is broad, and diagnostic capabilities are often limited, leading to empiric antimicrobials [5–7].

In East Africa, vector-borne diseases including chikungunya virus (CHIKV), dengue virus (DENV) and *Plasmodium* species (malaria) are endemic and account for a significant burden of AFI presentations, particularly among children [8–11]. Fortunately, rapid diagnostic tests (RDTs) for malaria are now accessible and allow for prompt diagnosis and administration of antiparasitics, but the diagnosis of arboviral infections remains largely clinical, and co-infections of malaria and arboviruses can also occur, further complicating the diagnostic picture [6,7,12,13].

Improving antimicrobial stewardship in LMICs requires a better understanding of AFI etiologies and antibiotic prescribing practices based on currently available diagnostics [14]. Our laboratory previously looked at clinical presentations and medical management in a cohort of over 5,000 Kenyan children presenting to outpatient clinics with fever between 2014 and 2017 and found that 68% received antibiotics despite less than a third receiving a diagnosis of a bacterial infection [9]. This data was accompanied by qualitative work using structured clinician interviews, where it was discovered that lack of resources, including diagnostics, and uneasiness with the primary diagnosis of viral illness lead to frequent empiric antibiotic prescriptions [15].

In this current study, we analyzed sick visits among adults and children presenting to two outpatient clinics in Western and Coastal Kenya with the goal of describing antibiotic prescribing trends, concordance of clinician diagnosis and appropriateness of antibiotics, and patient factors associated with antibiotic prescriptions. Our data encompasses visits between the end of 2019 and beginning of 2022, so we also aimed to explore differences in antibiotic prescribing practices before and during the COVID-19 pandemic in Kenya. By understanding antibiotic prescribing trends in low-resource clinical settings, we hope to build awareness about potential areas for meaningful interventions, including introduction of diagnostics and antimicrobial stewardship education and programs.

## Materials and methods

### Ethics statement

This retrospective, descriptive cohort study was part of a larger cohort study to determine arbovirus seroprevalence, seroconversion and factors which influence transmission in Western and Coastal Kenya (AI102918-08, PI: ADL). Written informed consent was obtained by all study participants, with parents/guardian consenting for children. This consent included use of clinical and laboratory data. Institutional Review Board approval for human subjects research was obtained from Stanford (#49683) and Technical University of Mombasa, Kenya (TUM ERC EXT/004/2019).

Participants in the larger cohort study were originally recruited house to house and provided baseline and follow-up serum samples with demographic and behavioral surveys completed by head of household. Participants received instructions to attend specific clinics if they experienced a fever during the study period, and these “sick visits” were subsidized for those enrolled in the larger cohort study. Participants were provided with an identification card to bring with them to the clinics, which identified them as enrolled in the study. Clinics had several medical officers based at them full time during the study period. There were two clinics included in the study, one in an urban area of Western Kenya and the other in an urban area in Coastal Kenya.

When any study participant presented to either of the two specified clinics endorsing a febrile illness, subjective and objective data from these visits was collected, including symptoms, vital signs and physical exam findings, treating clinician impressions, medications prescribed and any point of care laboratory testing that was completed during the visit (S1 File). Both clinics had malaria rapid diagnostic test (RDT) capabilities, which were performed onsite during the visit. Other testing available on site included uranalysis, hemoglobin, glucose, pregnancy testing, and HIV testing. CHIKV and DENV testing were performed at a later time as part of the larger R01 using a previously validated multiplex reverse transcription quantitative polymerase chain reaction (RT-qPCR) with pan-DENV and CHIKV primers [16]. These results were not available to the treating clinician at the time of the sick visit.

During each visit, the treating clinician was asked to select from a list of provisional diagnoses based on his/her assessment and the limited diagnostic testing he or she had available, with no limit to the number of diagnoses that could be selected. During our analysis, appropriateness of antibiotic prescription was determined based on clinician reported provisional diagnosis, with the following diagnoses considered possibly bacterial in etiology and therefore appropriate for an antibiotic prescription: bacterial infection, ear infection, eye infection, gastroenteritis, meningitis, peptic ulcer disease, pneumonia, skin infection, tonsillitis/pharyngitis, tuberculosis, typhoid, lower respiratory tract infection, urinary tract infection; and the following considered not typically appropriate for antibiotics: anemia, chikungunya, dengue, gastritis, HIV/AIDS, malaria, upper respiratory tract infection. If the only provisional diagnosis selected was either “unclear diagnosis at this time” or “other,” or if the question was not answered, appropriateness of antibiotic prescription was not defined.

Data collected was fully anonymized prior to being entered and stored on a REDCap database [17]. This anonymized data was exported into R statistical software (4.3.1) for analyses on 31 January 2023. In our analyses we included all sick visits that were recorded and entered into REDCap. Univariate analyses were performed using chi-square and OR (odds ratio) for categorical variables, or fisher’s exact when expected cell count was less than 5, and independent samples T-tests for continuous variables. Model selection for predicting if an encounter resulted in an antibiotic prescription was completed using multivariable logistic regression. Variable selection was based on prior epidemiologic studies and biologic plausibility [9]. Models were compared using Akaike Information Criterion (AIC), goodness of fit (R^2^), and number of excluded encounters. In all analyses, a p-value less than 0.05 was considered significant. Confidence intervals (CI) provided are 95%.

## Results

Between December 2019 and February 2022 there were 1,526 sick visit encounters documented among 1,059 people. Enrollment was continuous apart from April-June 2020 when the study was paused due to the COVID-19 pandemic. Majority of visits (79%) were recorded at the clinic in Western Kenya, and the remainder (21%) were from the clinic in Coastal Kenya. Median age at time of sick visit was 16 years (IQR 6–35 years), and 22% were under the age of 5 years. 666 persons (44%) reported onset of fever within 48 hours of presentation (Table 1).

**Table 1:** Demographic and clinical characteristics of sick visits by antibiotic prescription.

Variable		Antibiotic prescribed		p-value^1^
		Yes, N (row %) Total N = 1074	No, N (row %) Total N = 399	
Site	West	918 (77.5)	267 (22.5)	<0.0001
	Coast	156 (54.2)	132 (45.8)	
Sex	Female	654 (75.2)	216 (24.8)	0.019
	Male	420 (69.7)	183 (30.3)	
Age (mean, SD)		20.7 (17.9)	26.6 (17.9)	<0.0001^2^
Age category	≤18	618 (79.8)	156 (20.2)	<0.0001
	>18	456 (65.2)	243 (34.8)	
	<5	265 (83.6)	52 (16.4)	<0.0001
	≥5	809 (70.0)	347 (30.0)	
Pre vs during COVID-19 pandemic^3^	Q1 2020	46 (37.4)	77 (62.6)	<0.0001
	Q1 2021	286 (80.8)	68 (19.2)	
Season^4^	Rainy season	420 (75.9)	133 (24.1)	0.04
	Dry season	654 (71.1)	266 (28.9)	
Visit	Initial visit within month	1015 (72.1)	392 (27.9)	<0.0001
	Second visit within month	59 (89.4)	7 (10.6)	
Fever on exam	≥38 C	173 (75.6)	56 (24.5)	0.32
	<38 C	868 (72.3)	332 (27.7)	
Duration of fever	≥7 days	31 (66.0)	16 (34.0)	0.27
	<7 days	1041 (73.2)	381 (26.8)	
Patient reported symptoms	HEENT^5^	799 (82.2)	173 (17.8)	<0.0001
	Cardiorespiratory	824 (83.5)	163 (16.5)	<0.0001
	Gastrointestinal	286 (73.2)	105 (26.9)	0.006
	Musculoskeletal	599 (71.1)	243 (28.9)	0.07
	Neurologic	875 (73.5)	316 (26.5)	0.79
	Dermatologic	64 (78.0)	18 (22.0)	0.15
Abnormal physical exam findings	Overall exam	206 (87.0)	31 (13.0)	<0.0001
	HEENT	124 (83.8)	24 (16.2)	0.002
	Cardiorespiratory	40 (88.9)	5 (11.1)	0.01
	Gastrointestinal	29 (87.9)	4 (12.1)	0.05
	Musculoskeletal	20 (71.4)	8 (28.6)	0.84
	Neurologic	179 (87.3)	26 (12.7)	<0.0001
	Dermatologic	63 (79.7)	16 (20.3)	0.16
Malaria RDT^6^ result	Positive	178 (63.8)	101 (36.2)	0.039
	Negative	455 (70.7)	189 (29.3)	
Number of provisional diagnoses	0^7^	31 (21.4)	114 (78.6)	<0.001
	1	606 (74.3)	210 (25.7)	
	2	376 (85.8)	62 (14.2)	
	3	58 (81.7)	13 (18.3)	
	4	3 (100)	0 (0)	
Provisional diagnosis consistent with bacterial etiology^8^	Yes	489 (91.4)	46 (8.6)	<0.0001
	No	453 (73.7)	162 (26.3)	

^1^ Chi-square test, fisher’s exact when expected cell count <5.

^2^ Independent samples t-test.

^3^ Quarter 1 (Q1) considered pre-COVID-19 pandemic, Quarter 2 (Q2) during COVID-19 pandemic.

^4^ Rainy season considered March-May and November-December.

^5^ Head, eyes, ears, nose, throat.

^6^ Rapid diagnostic test.

^7^ Includes both “unclear diagnosis at this time” and question left blank.

^8^ Diagnoses considered possibly bacterial in etiology: Bacterial infection, ear infection, eye infection, gastroenteritis, meningitis, peptic ulcer disease, pneumonia, skin infection, tonsillitis/pharyngitis, tuberculosis, typhoid, lower respiratory tract infection, urinary tract infection.

The most commonly reported subjective symptoms among participants were neurologic (including confusion, dizziness and headache) in 81%, cardiorespiratory (including chest pain, cough, shortness of breath) in 67%, ear nose and throat symptoms (including rhinorrhea, sore throat, eye and ear pain or discharge) in 66%, gastrointestinal (including abdominal pain, constipation, diarrhea, nausea) in 60%, and skin (including pruritus, rash or sores) in 10%. Among those with recorded temperatures from the visit, 246/1478 (17%) were febrile with a temperature greater than or equal to 38 degrees Celsius.

Physical exam findings were documented as normal or abnormal by the treating clinician. 240/1513 (16%) were reported as having an overall abnormal physical exam. By system, 14% had a documented abnormal neurological exam (most common abnormality weakness), 10% an abnormal head, eyes, ears, nose and throat (HEENT) exam, 5% an abnormal dermatological exam, 3% an abnormal cardiorespiratory exam, and 2% an abnormal gastrointestinal exam.

Clinician prescription decisions were available for 1473/1526 (97%) of encounters, of which 1074 (73%) resulted in an antibiotic prescription. Prevalence of antibiotic prescriptions for children less than or equal to 18 years was 618/774 (80%) and for children less than 5 years was 265/317 (84%). Table 1 displays demographic and clinical characteristics of sick visit encounters stratified by antibiotic prescription. The most commonly prescribed antibiotics were amoxicillin (56%), metronidazole (10%), azithromycin (6%), ciprofloxacin (6%), and amoxicillin-clavulanic acid (5%). The full list of antibiotics prescribed is provided in S1 Table.

Malaria RDT was performed during 952 encounters, of which 289 (30%) were positive, including 172/516 (33%) in children. 64% of encounters with a confirmed malaria diagnosis still resulted an antibiotic prescription. CHIKV and DENV RT-qPCR testing, which was done later and outside of the clinic, was conducted on 967 encounter samples, with a DENV incidence of 3% in the total population and 3% in children, and a CHIKV incidence of 0.3% in the total population and 0.4% in children.

Treating clinicians were asked to select from a list of provisional diagnoses during each encounter. In 165/1526 (11%) of encounters, the clinician did not answer or selected “unclear diagnosis at this time.” The most selected provisional diagnoses were upper respiratory infection (647, 42%) and malaria (390, 26%). The full list of provisional diagnoses is provided in Table 2. 498/544 (92%) encounters where a provisional diagnosis could be considered bacterial in etiology resulted in an antibiotic prescription, while 453/615 (74%) of encounters where no bacterial diagnosis was provided resulted in an antibiotic prescription. Overall, 453/951 (48%) of encounters without a provisional diagnosis that could be bacterial in etiology resulted in an antibiotic prescription.

**Table 2:** Provisional diagnoses provided by treating clinician.

Provisional diagnosis^1^	N (%) total = 1526 encounters
Upper respiratory infection (URI)	647 (42.4)
Malaria	390 (25.6)
Tonsillitis/pharyngitis	173 (11.3)
Bacterial infection	118 (7.7)
Gastroenteritis	51 (3.3)
Typhoid	44 (2.7)
Urinary tract infection (UTI)	41 (2.7)
Pneumonia	39 (2.6)
Chikungunya	36 (2.4)
Skin infection	35 (2.3)
Lower respiratory tract infection (LRTI)	33 (2.2)
Peptic ulcer disease (PUD)	25 (1.6)
Gastritis	21 (1.4)
Eye infection	20 (1.3)
Dengue	18 (1.2)
Ear infection	16 (1.0)
Anemia	3 (0.2)
Tuberculosis (TB)	2 (0.1)
Meningitis	1 (0.1)
Sickle cell crisis	1 (0.1)
Other	239 (15.7)
“Unclear at this time” or left blank	165 (10.8)

^1^ Number of listed provisional diagnoses per encounter range 0–4 (median 1, IQR 1–2).

**Fig 1:**
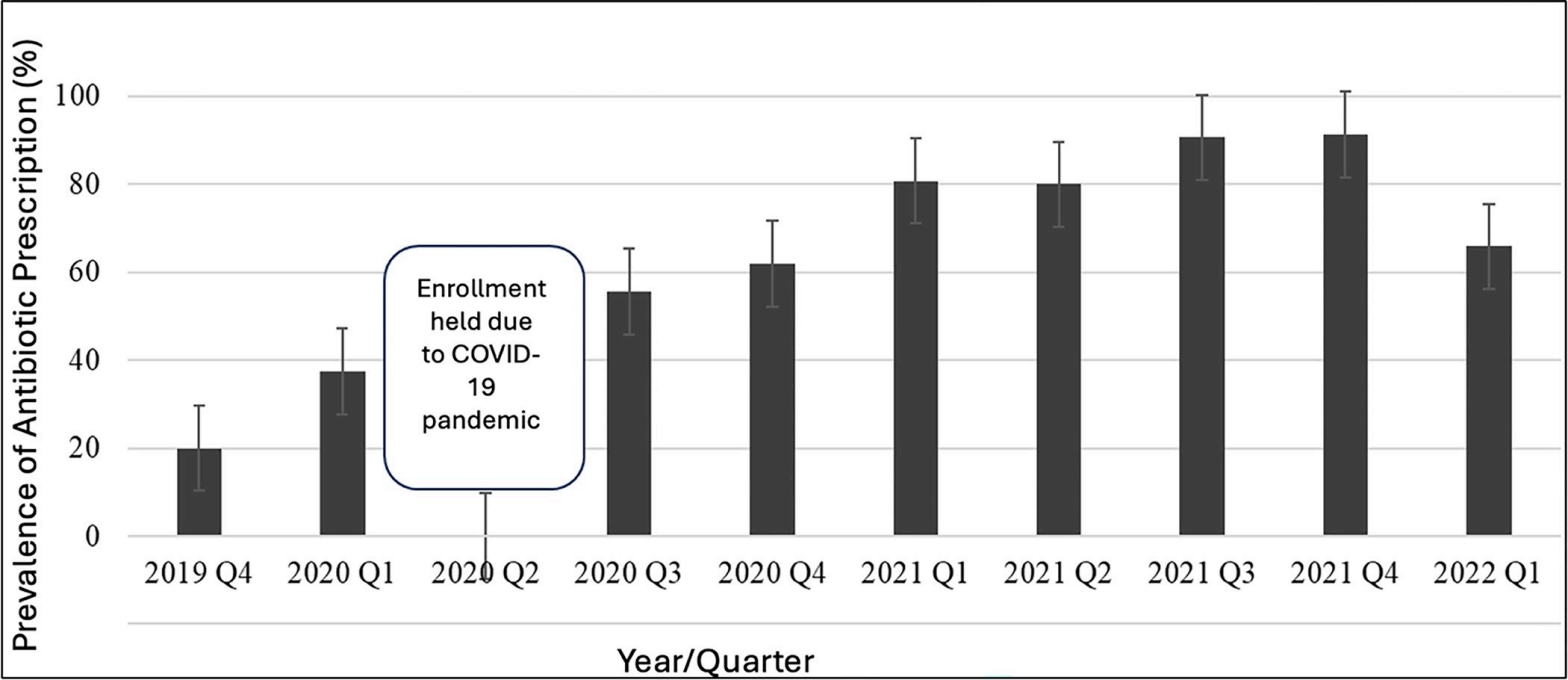
Prevalence of antibiotic prescription by year and quarter.

Antibiotic prescription prevalence by quarter is visually represented in Fig 1. We aimed to compare antibiotic prescriptions just prior to and during the COVID-19 pandemic. Specifically we compared quarter one (January, February, March) of 2020 (prior to significant SARS-CoV-2 transmission in Kenya) to the first quarter of 2021 [18]. Visits in quarter one of 2021 were at 7.0 times increased odds of receiving antibiotics compared with quarter one of 2020 in the univariate analysis (95% CI 4.5–11.1, p<0.0001).

The univariate analysis for variables associated with increased odds of antibiotic prescription are shown in Table 3. These were seeking care at the clinic in Western Kenya, female sex, younger age (both age < 5 and age ≤ 18, visits during the rainy season, being seen for a follow-up within the month, visits in quarter one of 2021 (during COVID-19 pandemic), a negative malaria RDT, and at least one provisional diagnosis that could be considered bacterial in etiology. Symptoms associated with increased odds of antibiotic prescription were HEENT, cardiorespiratory, and gastrointestinal. Abnormal physical exam findings associated with increased odds of antibiotic prescription were general exam, HEENT, cardiorespiratory, and neurologic. In our multivariable regression model, factors associated with increased odds of an antibiotic prescription were age less than or equal to 18 (OR 2.1, 95% CI 1.4–3.2), the clinic in Kisumu (OR 5.1, 95% CI 3.0–8.8), subjective endorsement of cardiorespiratory symptoms (OR 5.2, 95% CI 3.2–8.3), a negative malaria RDT (OR 4.0, 95% CI 2.5–6.8), and a provisional diagnosis that could be considered bacterial in etiology (OR 5.9, 95% CI 3.5–10.3). The R^2^ of this model was 0.68.

**Table 3:** Univariate and multivariable logistic regression of factors associated with antibiotic prescriptions.

Variable	Univariate			Multivariate		
	OR^1^	95% CI^2^	p-value^3^	OR	95% CI	p-value
West site	2.9	2.2–2.8	<0.0001	5.1	3.0–8.8	<0.0001
Female sex	1.3	1.1–1.7	0.02			
Age < 5	2.2	1.6–3.0	<0.0001			
Age≤18	2.1	1.7–2.7	<0.0001	2.1	1.4–3.2	<0.001
Quarter 1 2021^4^	7.0	4.5–11.1	<0.0001			
Rainy season^5^	1.3	1.0–1.6	0.04			
Return visit within the month	3.2	1.5–7.8	<0.001			
Temperature ≥38 Celsius	1.2	0.9–1.6	0.32			
Fever ≥7 days	0.7	0.4–1.3	0.28			
Patient reported symptoms						
HEENT^6^	3.9	3.1–5.0	<0.0001			
Cardiorespiratory	4.9	3.7–6.1	<0.0001	5.2	3.2–8.3	<0.0001
Gastrointestinal	1.7	1.3–2.1	<0.0001			
Musculoskeletal	0.8	0.6–1.0	0.07			
Neurologic	1.1	0.8–1.4	0.74			
Dermatologic	1.6	0.8–3.1	0.15			
Abnormal physical exam findings						
Overall exam	2.8	1.9–4.3	<0.0001			
HEENT	2.0	1.3–3.2	0.001			
Cardiorespiratory	3.0	1.3–8.8	0.01			
Gastrointestinal	2.6	1.0–9.2	0.05			
Musculoskeletal	0.9	0.4–2.2	0.82			
Neurologic	2.8	1.9–4.5	<0.0001			
Dermatologic	1.5	0.9–2.7	0.16			
Negative malaria RDT^7^	1.4	1.0–1.8	<0.0001	4.0	2.5–6.8	<0.0001
Provisional diagnosis consistent with bacterial etiology^8^	3.9	2.7–5.5	0.04	5.9	3.5–10.3	<0.0001

^1^ Odds ratio.

^2^ Confidence interval.

^3^ Odds ratio, or fisher’s exact when expected cell count <5.

^4^ During COVID-19 pandemic, compared to quarter 1 2020 (prior to COVID-19 pandemic).

^5^ March-May, November-December.

^6^ Head, eyes, ears, nose, throat.

^7^ Rapid diagnostic test.

^8^ Diagnoses considered possibly bacterial in etiology: Bacterial infection, ear infection, eye infection, gastroenteritis, meningitis, peptic ulcer disease, pneumonia, skin infection, tonsillitis/pharyngitis, tuberculosis, typhoid, lower respiratory tract infection, urinary tract infection.

## Discussion

In this retrospective, descriptive cohort study of sick visits at two outpatient clinics in Western and Costal Kenya, nearly three-quarters of encounters (73%) resulted in an antibiotic prescription despite less than half (48%) being associated with a clinician reported provisional diagnosis that could be considered bacterial in etiology. This finding was largely driven by antibiotics prescribed for the diagnosis of upper respiratory infection, which was the most common provisional diagnosis reported. We also found a seven-fold increased odds of antibiotic prescriptions when comparing visits before the COVID-19 pandemic reached Kenya (quarter one 2020) and after (quarter one 2021) in the univariate analysis. This trend of increased utilization of antibiotics has been seen worldwide during the COVID-19 pandemic [3].

In our study we also found higher rates of antibiotic prescriptions when looking specifically at children 18 years and under (80%) and children less than 5 years (84%) with age remaining a significant predictor of antibiotic prescription in our multivariable model. Nearly all children who were presenting for the second time within the same month received an antibiotic prescription, and children who received one or more provisional diagnoses were more likely to receive antibiotics than when the provider listed unknown or other (S2). We hypothesis that these findings might be explained by parent or guardian expectations and clinician training or comfort in the care pediatric patients.

Another important finding from our study is that while a negative malaria RDT was associated with increased odds of antibiotic prescription in our multivariable model, we found that 64% of persons with a positive malaria RDT also received antibiotics, and there was no significant association between RDT results and antibiotic prescription when looking specifically at adults greater than 18 years of age (S2 and S3 Tables). This suggests that even when malaria diagnostics are available, questions regarding co-infections or need for additional antimicrobials apart from anti-malarial medications still exist, especially among adults. This finding may again also be partially explained by patient expectations.

Compared to the 2014–2017 Kenyan cohort of children with undifferentiated febrile illness, we saw an increase in the prevalence of antibiotic prescriptions in children less than or equal to 18 years (68% vs 80%) and children under 5 years (73% vs 84%), which has also been observed in other LMICs [9,19]. Several of the factors associated with antibiotic prescriptions identified in this original studying, including a negative malaria test, a more urban (Western) clinic and a provisional bacterial diagnosis remained significant factors predicting antibiotic prescriptions [9].

Our study has several limitations. First, our analysis was limited to what was recorded by the treating clinician, which consisted mostly of check boxes without space for explanations, so details that may have influenced prescribing patterns may not be accounted for, such as additional history of illness, patient or family expectations, ability to follow-up, etc. Second, a high number of encounters (239, 18%), were given a diagnosis of “other,” so we were unable to draw conclusions about the appropriateness of antibiotic prescriptions. Next, the study sites were limited to only 2 clinics, each with only a few clinical officers, so the generalizability of our findings both within Kenya as well as to other LMICs may be limited.

Our findings have important implications for this population. High rates of antibiotic use directly contribute to increase rates of antibiotic resistance that we are seeing worldwide and particularly in sub-Saharan Africa, which has significant downstream effects related to cost, morbidity and mortality [1]. To improve antibiotic stewardship in LMICs, interventions need to target both community members as well as clinicians. The Integrated Management of Child Health (IMCI) guidelines are still widely taught to assist clinicians in diagnosis and treatment in children under 5 years in resource limited settings, but studies have shown they are not always followed [20]. In our study, majority of children still received antibiotics for a URI, which is not recommended by IMCI. Additionally, some of the diagnostic criteria for bacterial infections may be oversensitive, such as associating cough with pneumonia when it may be due to asthma [21]. In older children and adults, there are no widely available set of guidelines, so diagnoses and recommendations must be made based on largely subjective information provided to the clinician in combination with physical exam. In the absence of diagnostics, clinicians may be swayed to the conservative approach of providing empiric antibiotics. Point of care diagnostics, including respiratory viral panels, SARS-Co-V2 and influenza antigen testing, chest x-rays are rarely available in outpatient health centers in LMICs but have the potential to provide more clarity to clinicians when formulating a diagnosis, which can lessen the need for empiric antibiotics. Patient factors also drive antibiotic use. The ease at which people can purchase antibiotics over the counter without a prescription, combined with lack of awareness regarding antibiotic properties, indications and resistance pathogens leads to high rates of self-administration [22,23]. Additionally, patient expectations have been shown to influence provider prescribing [24].

We feel that our findings, when compared to our prior cohort study from several years ago, show a concerning increase in antibiotic prescriptions, many of which may be inappropriate based on provisional diagnosis, and continue to highlight the need for action at both the provider and community level. Future studies need to examine how interventions such as provider stewardship education, point of care testing and community education improve appropriate antibiotic use specifically in LMICs. AMR is an urgent public health threat globally, and tools to curb antibiotic misuse that can be implemented in LMICs are needed. Small changes at individual and community level have the potential to lead to country-wide improvements in antibiotic regulation and stewardship program funding.

## Supporting information

S1 File Sick visit survey. (PDF)

S1 Table Antibiotics prescribed during sick visits. (DOCX)

S2 Table Demographic and clinical characteristics of sick visits by antibiotic prescription for children ≤18 of age. ^1^ Chi-square test, fisher’s exact when expected cell count <5. ^2^ Independent samples t-test. ^3^ Quarter 1 (Q1) considered pre-COVID-19 pandemic, Quarter 2 (Q2) during COVID-19 pandemic. ^4^ Rainy season considered March-May and November-December. ^5^ Head, eyes, ears, nose, throat. ^6^ Rapid diagnostic test. ^7^ Includes both “unclear diagnosis at this time” and question left blank. ^8^ Diagnoses considered possibly bacterial in etiology: Bacterial infection, ear infection, eye infection, gastroenteritis, meningitis, peptic ulcer disease, pneumonia, skin infection, tonsillitis/pharyngitis, tuberculosis, typhoid, lower respiratory tract infection, urinary tract infection.
(DOCX)

S3 Table Demographic and clinical characteristics of sick visits by antibiotic prescription for adults >18 years of age. ^1^ Chi-square test, fisher’s exact when expected cell count <5. ^2^ Independent samples t-test. ^3^ Quarter 1 (Q1) considered pre-COVID-19 pandemic, Quarter 2 (Q2) during COVID-19 pandemic. ^4^ Rainy season considered March-May and November-December. ^5^ Head, eyes, ears, nose, throat. ^6^ Rapid diagnostic test. ^7^ Includes both “unclear diagnosis at this time” and question left blank. ^8^ Diagnoses considered possibly bacterial in etiology: Bacterial infection, ear infection, eye infection, gastroenteritis, meningitis, peptic ulcer disease, pneumonia, skin infection, tonsillitis/pharyngitis, tuberculosis, typhoid, lower respiratory tract infection, urinary tract infection.
(DOCX)
