# Supplementary material for: Antibiotic prescribing patterns at outpatient clinics in Western and Coastal Kenya
Source: PLOS Glob Public Health. 2025 Jan 3;5(1):e0004109. doi: 10.1371/journal.pgph.0004109 (PMC11698307; doi:10.1371/journal.pgph.0004109)
Supplement: S1 File — (PDF) [file pgph.0004109.s001.pdf]

Sick Visit Survey

Record ID ______________________

How long have you been sick?

FOR THE CLINICAL OFFICER: Please enter how long the respondent has BEEN SICK. This means total time feeling unwell and NOT just the time with fever. Not all three fields need to be filled out. For example, if the participant says "2 days," just put "2" in the "days" field and leave the other fields blank. If "3 months," put "3" in the "months" field.

Days ______________________

Weeks ______________________

Months ______________________

How long have you had a fever?

FOR THE CLINICAL OFFICER: Please enter how long the respondent has had a fever. Not all three fields need to be filled out. For example, if the participant says "2 days," just put "2" in the "days" field and leave the other fields blank. If "3 months," put "3" in the "months" field.

Days ______________________

Weeks ______________________

Months ______________________

What symptoms have you had during this illness?

Instructions for clinical officer: ask the participant about EACH specific organ system listed below. If they say they had a problem with that organ system, check the box and then you will be prompted to specify exactly what the problem was.

	Yes	No	Unsure/Don't Know
PROBLEMS WITH HEAD, BRAIN, OR NECK	○	○	○

Confusion	○	○	○
Dizziness	○	○	○
Headache	○	○	○
Neck swelling	○	○	○
Seizures	○	○	○
Stiff neck	○	○	○
PROBLEMS WITH EYES, EARS, NOSE, THROAT	○	○	○
Eye discharge	○	○	○
Eye pain	○	○	○
Eye redness	○	○	○
Yellow eyes	○	○	○
Blurry vision	○	○	○
Ear discharge	○	○	○
Ear pain	○	○	○
Runny nose	○	○	○
Sore throat	○	○	○
PROBLEMS WITH BREATHING OR CHEST	○	○	○
Chest Pain	○	○	○
Cough	○	○	○
Difficulty breathing	○	○	○
STOMACH PROBLEMS	○	○	○
Abdominal Pain	○	○	○
Constipation	○	○	○
Diarrhea	○	○	○
Nausea or Vomiting	○	○	○
PROBLEMS WITH MUSCLES, JOINTS, OR EXTREMITIES	○	○	○
Joint pain	○	○	○
Joint stiffness	○	○	○
Joint swelling	○	○	○
Muscle pain	○	○	○
Back pain	○	○	○
Flank pain	○	○	○
Numbness in part of the body	○	○	○
Weakness in part of the body	○	○	○
PROBLEMS WITH SKIN OR BLOOD	○	○	○
Abnormal bleeding	○	○	○
Itching	○	○	○

Rashes	◯	◯	◯
Sores	◯	◯	◯
Dark urine	◯	◯	◯
OTHER	◯	◯	◯

If "other," what are your current symptoms? ______________________________

Have you had any abnormal bleeding?
◯ Yes
◯ No

If yes, where?
◯ Stool
◯ Urine
◯ Gums
◯ Menstrual
◯ Vomit
◯ Other

If other, please specify ______________________________

Have you taken any medications for your current illness during the past two weeks?
◯ Yes
◯ No

Which of the following medications have you taken in the past two weeks for your current illness?

	Yes	No
ANTIMALARIAL	◯	◯
Antimalarial, unknown type	◯	◯
Artesunate (IV)	◯	◯
Artemether/lumafantrine	◯	◯
Dihydroartemisinin-piperaquine	◯	◯
Primaquin	◯	◯
Quinine (IV or PO)	◯	◯
Other antimalarial (write-in field will be found below)	◯	◯
ANTIBIOTIC	◯	◯
Antibiotic, unknown type	◯	◯
Amoxicillin	◯	◯
Augmentin	◯	◯
Azithromycin	◯	◯
Ceftriaxone	◯	◯
Ciprofloxacin	◯	◯
Doxycycline	◯	◯

Gentamicin	◯	◯
Metronidazole	◯	◯
Penicillin	◯	◯
Other antibiotic (write-in field will be found below)	◯	◯
ANTIPARASITIC	◯	◯
Antiparasitic, unknown type	◯	◯
Albendazole	◯	◯
Ivermectin	◯	◯
Praziquantel	◯	◯
Other antiparasitic (write-in field will be found below)	◯	◯
OTHER MEDICATION FROM CHEMIST OR PHARMACIST	◯	◯
Unknown type	◯	◯
Antihistamine	◯	◯
Ibuprofen	◯	◯
Paracetamol	◯	◯
Other medication (write-in field will be found below)	◯	◯

If other, what other antimalarial have you taken in the past two weeks? ________________________________

If other, what other antibiotic have you taken in the past two weeks? ________________________________

If other, what other antiparasitic have you taken in the past two weeks? ________________________________

If other, what other pharmacy medication have you taken in the past two weeks? ________________________________

Have you sought care for this fever prior to this visit?
- ◯ Yes
- ◯ No

If yes, what kind of care?
- ☐ Clinic or hospital
- ☐ Community health worker outside a clinic or hospital
- ☐ Pharmacy or chemist
- ☐ Care from a family member or acquaintance NOT trained as healthcare worker
- ☐ Traditional healer
- ☐ Other

If "other," what kind of care did you receive? ________________________________

If a clinic or hospital, which clinic or hospital did you go to?	☐ Obama Childrens Hospital<br>☐ Likoni Health Center<br>☐ Jaramogi Odinga Referral Hospital<br>☐ Ukunda Medical Center<br>☐ Kisumu Hospital<br>☐ Msambweni County Referral Hospital<br>☐ Migosi Subcounty Referral Hospital<br>☐ Kwale Subcounty Hospital<br>☐ Other
If other, what clinic or hospital did you go to?	__________________________________
Has a clinical officer from this study already seen you for this fever?	◯ Yes<br>◯ No

Medical History

Do you take any medication on a regular basis?	◯ Yes<br>◯ No
If yes, which ones?	☐ Antacids<br>☐ Anticonvulsant<br>☐ Antidepressant/Antipsychotic<br>☐ Antifungals<br>☐ Antihypertensive<br>☐ Antiretrovirals<br>☐ Diabetes medication<br>☐ Family planning<br>☐ Tuberculosis medication<br>☐ Other
Which other medication do you take on a regular basis?	__________________________________
Are you currently pregnant?	◯ Yes<br>◯ No
When is your anticipated due date for your baby?	__________________________________

Vital Signs

Heart rate, in beats/minute	__________________________________<br>(Beats/minute)
Respiratory Rate, in breaths/minute	__________________________________<br>(Breaths/minute)
Systolic blood pressure, in mmHG	__________________________________<br>(mmHg)

Diastolic blood pressure, in mmHg

____
(mmHg)

Oxygen Saturation, in percent

____
(%)

Anthropometric Measures

FOR INTERVIEWER: Below you will find anthropometric fields for height, weight, mid-upper arm circumference (MUAC), and head circumference.

Each measurement should be taken multiple times:

Height and Weight should be measured twice

MUAC and head circumference should be measured three times

An alert will appear if you have entered values that have too much variation

Participant Height - Measurement 1 (cm)

____

Participant Height - Measurement 2 (cm)

____

WARNING TO INTERVIEWER: Your height measurements are greater than >1cm apart. Please retake the weight measurements

Participant Length - Measurement 1 (cm)

____

Participant Length - Measurement 2 (cm)

____

WARNING TO INTERVIEWER: Your length measurements are greater than >1cm apart. Please retake the weight measurements

Participant Weight - Measurement 1 (Kg)

____

Participant Weight - Measurement 2 (Kg)

____

WARNING TO INTERVIEWER: Your weight measurements are greater than >0.5kg apart. Please retake the weight measurements

Participant BMI

____

Head Circumference - Measurement 1 (cm) ______________________

Head Circumference - Measurement 2 (cm) ______________________

Head Circumference - Measurement 3 (cm) ______________________

WARNING TO INTERVIEWER: Your head circumference measurements are greater than >0.5cm apart. Please retake the head circumference measurements

Mid-upper arm circumference (MUAC) - Measurement 1 (cm) ______________________

Mid-upper arm circumference (MUAC) - Measurement 2 (cm) ______________________

Mid-upper arm circumference (MUAC) - Measurement 3 (cm) ______________________

WARNING TO INTERVIEWER: Your MUAC measurements are greater than >0.5cm apart. Please retake the MUAC measurements

Visual Acuity

Is the patient wearing eyewear?
- ◯ Yes
- ◯ No

Left eye visual acuity ______________________

NOTE TO CLINICAL OFFICER: For visual acuity, record ONLY THE BOTTOM number from the chart.

Right eye visual acuity ______________________

NOTE TO CLINICAL OFFICER: For visual acuity, record ONLY THE BOTTOM number from the chart.

Physical Examination

Instructions for Clinical Officers: Check whether an organ system is normal or abnormal. If abnormal, a list will pop up so you can document the abnormalities.

GENERAL APPEARANCE
- ◯ Normal
- ◯ Abnormal

Abnormality in general appearance
- ☐ In distress
- ☐ Cachectic (wasted)
- ☐ Confused
- ☐ Physical deformity
- ☐ Other

Other overall abnormality ______________________

HEAD, FACE, NECK	◯ Normal<br>◯ Abnormal
Head, face, & neck abnormality	☐ Eyes red<br>☐ Eyes yellow<br>☐ Eye discharge<br>☐ Ear discharge<br>☐ Oral lesions<br>☐ Stiff neck<br>☐ Throat redness or exudate<br>☐ Other
Other face, neck, and neck abnormality	__________________________________
CARDIOVASCULAR AND RESPIRATORY SYSTEM	◯ Normal<br>◯ Abnormal
Cardiovascular OR respiratory system abnormality	☐ Abnormal heart rate or rhythm<br>☐ Unequal air entry<br>☐ Respiratory distress<br>☐ Wheezing<br>☐ Other
Other cardiovascular and respiratory system abnormality	__________________________________
GASTROINTESTINAL	◯ Normal<br>◯ Abnormal
Gastrointestinal abnormality	☐ Palpable liver<br>☐ Palpable spleen<br>☐ Tenderness with palpitation<br>☐ Other
Other gastrointestinal abnormality	__________________________________
EXTREMITIES	◯ Normal<br>◯ Abnormal
Extremities abnormality	☐ Edema<br>☐ Joint redness<br>☐ Joint swelling<br>☐ Other
Other extremities abnormality	__________________________________
NEUROLOGIC	◯ Normal<br>◯ Abnormal
Neurological abnormalities	☐ Confusion<br>☐ Numbness<br>☐ Weakness<br>☐ Other

Other neurological abnormalities	__________________________
DERMATOLOGIC OR HEMATOLOGIC	◯ Normal<br>◯ Abnormal
Dermatologic abnormality	☐ Rash<br>☐ Sores<br>☐ Bleeding<br>☐ Other
Other dermatologic abnormality	__________________________

Risk Factors Questions

Do you think it is possible, based on the history and physical examination, that this participant MIGHT have acute chikungunya or dengue?<br><br>NOTE TO THE CLINICAL OFFICERS:<br>1. For chikungunya or dengue to be HIGHLY UNLIKELY causes of the participant's fever, a focal and discrete alternative causes of fever should be identified (i.e otitis media, appendicitis, post-operative infection, etc).<br>2. If chikungunya or dengue are POSSIBLE, phlebotomy should be performed so testing for acute infection can be performed.	◯ Yes, chikungunya or dengue is POSSIBLE<br>◯ No, chikungunya or dengue is HIGHLY UNLIKELY
Have you been in contact with other people with fever in the last 7 days?	◯ Yes<br>◯ No<br>◯ Don't Know
If yes, who have you been in contact with that had a fever?	☐ Family member<br>☐ Neighbor<br>☐ Community Member (not a neighbor)<br>☐ Someone at work or school<br>☐ Other
If other, who else have you been in contact with that had a fever?	__________________________
Are your vaccinations up to date?	◯ Yes<br>◯ No
If no, which vaccinations are you behind on?	________________________________
Have you ever been vaccinated against yellow fever?	◯ Yes<br>◯ No<br>◯ Don't Know

If yes, when were you vaccinated for yellow fever?

If only the year is known, please use January 1st as the date.

Ex: 01Jan2010

________________________ (Format: DD - Month (in Letters) - YYYY (EX: 20Feb2019))

INSTRUCTIONS FOR CLINICAL OFFICER: Was the date of the yellow fever vaccination verified by vaccination card or other document?

◯ Yes
◯ No

Mosquito Exposure

Have you been bitten by mosquitoes in the last 2 weeks?

◯ Yes
◯ No
◯ Don't Know

If yes, when?

☐ Daytime
☐ Nighttime
☐ Don't know

Did you sleep under a mosquito net last night?

◯ Yes
◯ No

How often do you sleep under a mosquito net?

◯ Always
◯ Sometimes
◯ Rarely
◯ Never

Travel

In the past month, have you traveled more than 10km away from your home?

◯ Yes
◯ No
◯ Don't Know

What was your travel destination?

INSTRUCTIONS FOR DATA COLLECTOR: Try to get as close to the exact location as possible by asking whatever would be needed for you to pinpoint the location on a map. For instance, if the participant gives the name of a town and you know the place, you can stop there. If they cannot give a town name, try to get the name of the area or region.

Neighborhood/estate ________________________

City/town ________________________

County/region ________________________

Country (if other than Kenya) ________________________

How many nights did you spend there? ________________________

Did you travel to a second destination?	◯ Yes<br>◯ No
What was your second travel destination?<br><br>INSTRUCTIONS FOR DATA COLLECTOR: As above, try to get as close to the exact location as possible.	
Neighborhood/estate	______________________
City/town	______________________
County/region	______________________
Country (if other than Kenya)	______________________
How many nights did you spend there?	______________________
Did you travel to a third destination?	◯ Yes<br>◯ No
What was your third travel destination?<br><br>INSTRUCTIONS FOR DATA COLLECTOR: As above, try to get as close to the exact location as possible.	
Neighborhood/estate	______________________
City/town	______________________
County/region	______________________
Country (if other than Kenya)	______________________
How many nights did you spend there?	______________________
Were you bitten by mosquitoes during your trip?	◯ Yes<br>◯ No<br>◯ Don't Know
Did you get sick during your trip?	◯ Yes<br>◯ No

Travel

What symptoms did you have during the illness you got while traveling?

	Yes	No	Unsure/Don't Know
PROBLEMS WITH HEAD, BRAIN, OR NECK	○	○	○
Confusion	○	○	○
Dizziness	○	○	○
Headache	○	○	○
Neck swelling	○	○	○
Seizures	○	○	○
Stiff neck	○	○	○
PROBLEMS WITH EYES, EARS, NOSE, OR THROAT	○	○	○
Eye discharge	○	○	○
Eye pain	○	○	○
Eye redness	○	○	○
Yellow eyes	○	○	○
Ear discharge	○	○	○
Ear pain	○	○	○
Runny nose	○	○	○
Sore throat	○	○	○
PROBLEMS WITH CHEST OR BREATHING	○	○	○
Chest Pain	○	○	○
Cough	○	○	○
Difficulty breathing	○	○	○
STOMACH PROBLEMS	○	○	○
Abdominal Pain	○	○	○
Constipation	○	○	○
Diarrhea	○	○	○
Nausea or Vomiting	○	○	○
PROBLEMS WITH MUSCLES, JOINTS, OR EXTREMITIES	○	○	○
Joint pain	○	○	○
Joint stiffness	○	○	○
Joint swelling	○	○	○
Muscle pain	○	○	○
Numbness in part of the body	○	○	○
Weakness in part of the body	○	○	○

PROBLEMS WITH SKIN OR BLOOD	◯	◯	◯
Abnormal bleeding	◯	◯	◯
Itching	◯	◯	◯
Rashes	◯	◯	◯
Sores	◯	◯	◯
OTHER	◯	◯	◯

If you had other symptoms during your travels, what were they? __________________________

Did you receive a diagnosis for the illness that occurred during your travels?
- ◯ Yes
- ◯ No

What diagnosis did you receive?
- ☐ Unclear diagnosis
- ☐ Anemia
- ☐ Bacterial infection
- ☐ Chikungunya
- ☐ Dengue
- ☐ Ear infection
- ☐ Eye infection
- ☐ Gastritis
- ☐ Gastroenteritis
- ☐ HIV/AIDS
- ☐ Intestinal parasite(s)
- ☐ Malaria
- ☐ Meningitis
- ☐ Peptic ulcer
- ☐ Pneumonia
- ☐ Schistosomiasis
- ☐ Sickle cell crisis
- ☐ Skin infection
- ☐ Tonsilitis/Pharyngitis
- ☐ Tuberculosis
- ☐ Typhoid
- ☐ Upper respiratory tract infection ('cold')
- ☐ Lower respiratory tract infection ('pneumonia')
- ☐ Urinary Tract Infection
- ☐ Other

If other, what diagnosis did you receive for this illness? __________________________

We notice that there may be rift valley fever virus (RVFV) in this area. I am now going to ask you some questions to assess your risk of disease exposure

Livestock

Have you eaten raw meat in the last two weeks?
- ◯ Yes
- ◯ No

If yes, what animal? __________________________

Have you consumed raw milk in the last two weeks?
- ◯ Yes
- ◯ No

Have you consumed any raw animal blood in the past 2 weeks?	◯ Yes<br>◯ No
Have you consumed any raw fermented milk in the past 2 weeks?	◯ Yes<br>◯ No
Have you assisted an animal giving birth in the past 2 weeks?	◯ Yes<br>◯ No
If yes, what animal?	__________________________
Have you participated in butchering an animal in the past 2 weeks?	◯ Yes<br>◯ No
If yes, what animal?	☐ Cows<br>☐ Goats<br>☐ Sheep<br>☐ Camels<br>☐ Dogs<br>☐ Cats<br>☐ Buffaloes<br>☐ Antelope
If yes, what protective gear did you use while butchering?	☐ Gloves<br>☐ Mask<br>☐ Boots<br>☐ Eye protection<br>☐ Aprons<br>☐ White laboratory coat<br>☐ Other<br>☐ None
What other protective gear?	__________________________
Were you the one that cut this animals' throat?	◯ Yes<br>◯ No
Do you have animals?	◯ Yes<br>◯ No
Have any of your animals bled abnormally over the past 2 weeks?	◯ Yes<br>◯ No
What species?	__________________________
How many animals?	__________________________
Have any of your animals had an abortion over the past 2 weeks?	◯ Yes<br>◯ No
What species?	__________________________

How many animals?

____

Samples collected

FOR CO: Please enter any comments on the blood sample collected if anything unusual occurred (some examples: could not obtain sample; small amount obtained).

____

Rapid Diagnostic Test Results

Did you perform an RDT for malaria?

- ◯ Yes
- ◯ No

Results of rapid diagnostic test (RDT) for Malaria

- ◯ Positive
- ◯ Negative
- ◯ Inconclusive
- ◯ Not done

Photo of Malaria RDT, if inconclusive

Other Test Results

Did you perform or order other laboratory tests?

NOTE FOR CO: This means any tests besides those for chikungunya, dengue, and malaria.

- ◯ Yes, from this healthcare facility's lab
- ◯ Yes, from an outside lab
- ◯ No

If yes, which of the following tests?

- ☐ Glucose
- ☐ Hemoglobin
- ☐ Urinalysis
- ☐ Pregnancy test
- ☐ HIV test
- ☐ Hepatitis B antibody
- ☐ H pylori
- ☐ Malaria smear
- ☐ Typhoid test
- ☐ Other

Glucose result

____

Hemoglobin result

____

Urinalysis results

- ◯ Normal
- ◯ Abnormal

Comment if urinalysis results is abnormal

____

Pregnancy results

- ◯ Positive
- ◯ Negative
- ◯ other

Please describe pregnancy test results

____

HIV test results	◯ Positive<br>◯ Negative<br>◯ Other
Other HIV results	__________________________
Hepatitis B antibody test result	◯ Positive<br>◯ Negative<br>◯ Other
Other Hepatitis B results	__________________________
H pylori test result	◯ Positive<br>◯ Negative<br>◯ Other
Other H Pylori test results	__________________________
Malaria smear test result (at the health centre)	◯ Positive<br>◯ Negative<br>◯ Other
Other malaria test results	__________________________
Typhoid test result	◯ Positive<br>◯ Negative<br>◯ Other
Other typhoid test results	__________________________
If other, what other laboratory tests did you order?	__________________________

Sick Visit Diagnosis

What is your provisional diagnosis for this participant?<br><br>NOTE FOR CO: This refers to your assessment of the participant's diagnosis given the information you have at this time (your clinical assessment and any test results that are already available).	☐ Unclear diagnosis at this time<br>☐ Anemia<br>☐ Bacterial infection<br>☐ Chikungunya<br>☐ Dengue<br>☐ Ear infection<br>☐ Eye infection<br>☐ Gastritis<br>☐ Gastroenteritis<br>☐ HIV/AIDS<br>☐ Intestinal parasite(s)<br>☐ Malaria<br>☐ Meningitis<br>☐ Peptic ulcer<br>☐ Pneumonia<br>☐ Schistosomiasis<br>☐ Sickle cell crisis<br>☐ Skin infection<br>☐ Tonsilitis/Pharyngitis<br>☐ Tuberculosis<br>☐ Typhoid<br>☐ Upper respiratory tract infection<br>☐ Lower respiratory tract infection<br>☐ Urinary Tract Infection<br>☐ Other
Other provisional diagnosis	__________________________

Medications Prescribed

	Yes	No
ANTIMALARIAL	○	○
Artesunate (IV)	○	○
Artemether/lumafantrine	○	○
Dihydroartemisinin-piperaquine	○	○
Primaquin	○	○
Quinine (IV or PO)	○	○
Other antimalarial (write-in field will be found below)	○	○
ANTIBIOTIC	○	○
Amoxicillin	○	○
Augmentin	○	○
Azithromycin	○	○
Ceftriaxone	○	○
Ciprofloxacin	○	○
Doxycycline	○	○
Gentamicin	○	○
Metronidazole	○	○

Penicillin	○	○
Other antibiotic (write-in field will be found below)	○	○
ANTIPARASITIC	○	○
Albendazole	○	○
Ivermectin	○	○
Praziquantel	○	○
Other antiparasitic (write-in field will be found below)	○	○
OTHER MEDICATION	○	○
Antihistamine	○	○
Ibuprofen	○	○
Paracetamol	○	○
Other medication (write-in field will be found below)	○	○

If other, what other antimalarial have you taken in the past two weeks? ______________________________

If other, what other antibiotic have you taken in the past two weeks? ______________________________

If other, what other antiparasitic have you taken in the past two weeks? ______________________________

If other, what other pharmacy medication have you taken in the past two weeks? ______________________________

Visit Outcome/Follow-up

Outcome of Sick Visit
- ○ No follow-up
- ○ Follow-up
- ○ Referred to hospital
- ○ Other

Other outcome ______________________________

If referred to a hospital, where was the participant sent?
- ○ Obama Children's Hospital
- ○ Jaramogi Odinga Referral Hospital
- ○ Migosi Subcounty Hospital
- ○ Other

If referred to a hospital, where was the participant sent?
- ○ Msambweni County Referral Hospital
- ○ Kwale Sub-County Hospital
- ○ Other

Other hospital where sent ______________________________

Question	Response
When is follow-up scheduled to occur?	◯ Tomorrow<br>◯ In two days<br>◯ In three days<br>◯ In one week<br>◯ Other
Other when follow-up is scheduled to occur	__________________________________
Where is follow-up scheduled to occur?	◯ CO will go to participant's home<br>◯ Participant will go to clinic<br>◯ Other
Other place follow-up scheduled to occur?	__________________________________
Was this sick visit an Ajua survey referral?	◯ Yes<br>◯ No
