# Supplementary material for: Antibiotic prescribing patterns at outpatient clinics in Western and Coastal Kenya
Source: PLOS Glob Public Health. 2025 Jan 3;5(1):e0004109. doi: 10.1371/journal.pgph.0004109 (PMC11698307; doi:10.1371/journal.pgph.0004109)
Supplement: S1 Table — (DOCX) [file pgph.0004109.s002.docx]

**S1 Table.** **Antibiotics prescribed during sick visits.**

| **Antibiotic** | **N(%)** |
| --- | --- |
| Amoxicillin | 596 (51.7) |
| Metronidazole | 105 (9.1) |
| Azithromycin | 66 (5.7) |
| Ciprofloxacin | 65 (5.6) |
| Amoxicillin-Clavulanic Acid | 52 (4.5) |
| Ceftriaxone | 13 (1.1) |
| Penicillin | 13 (1.1) |
| Gentamicin | 2 (0.2) |
| Doxycycline | 1 (0.1) |
| Other | 240 (20.8) |
