# Supplementary material for: Antibiotic prescribing patterns at outpatient clinics in Western and Coastal Kenya
Source: PLOS Glob Public Health. 2025 Jan 3;5(1):e0004109. doi: 10.1371/journal.pgph.0004109 (PMC11698307; doi:10.1371/journal.pgph.0004109)
Supplement: S2 Table — 1 Chi-square test, fisher’s exact when expected cell count <5. 2 Independent samples t-test. 3 Quarter 1 (Q1) considered pre-COVID-19 pandemic, Quarter 2 (Q2) during COVID-19 pandemic. 4 Rainy season considered March-May and November-December. 5 Head, eyes, ears, nose, throat. 6 Rapid diagnostic test. 7 Includes both “unclear diagnosis at this time” and question left blank. 8 Diagnoses considered possibly bacterial in etiology: Bacterial infection, ear infection, eye infection, gastroenteritis, meningitis, peptic ulcer disease, pneumonia, skin infection, tonsillitis/pharyngitis, tuberculosis, typhoid, lower respiratory tract infection, urinary tract infection. (DOCX) [file pgph.0004109.s003.docx]

**S2 Table.** **Demographic and clinical characteristics of sick visits by antibiotic prescription for children ≤18 of age.**

| **Variable** | | **Antibiotic prescribed** | | **p-value^1^** |
| --- | --- | --- | --- | --- |
|  |  | **Yes, N (row %)**  **Total N=618** | **No, N (row %)**  **Total N=156** |  |
| Site | West | 520 (84.6) | 95 (15.4) | <0.0001 |
|  | Coast | 98 (61.6) | 61 (38.4) |  |
| Sex | Female | 328 (82.6) | 69 (17.4) | 0.048 |
|  | Male | 290 (76.9) | 87 (23.1) |  |
| Age | <5 | 265 (83.6) | 52 (16.4) | 0.03 |
|  | ≥5 | 353 (77.2) | 104 (22.8) |  |
| Pre vs during COVID-19 pandemic^3^ | Q1 2020 | 29 (48.3) | 31 (51.7) | <0.0001 |
|  | Q1 2021 | 176 (88.4) | 23 (11.6) |  |
| Season^4^ | Rainy season | 226 (81.3) | 52 (18.7) | 0.451 |
|  | Dry season | 392 (79.0) | 104 (21.0) |  |
| Visit | Initial visit within month | 577 (79.0) | 153 (21.0) | 0.02 |
|  | Second visit within month | 41 (93.2) | 3 (6.8) |  |
| Fever on exam | ≥38 C | 144 (78.3) | 40 (21.7) | 0.589 |
|  | <38 C | 457 (80.5) | 111 (19.5) |  |
| Duration of fever | ≥7 days | 8 (61.5) | 5 (38.5) | 0.19 |
|  | <7 days | 610 (80.2) | 151 (19.8) |  |
| Patient reported symptoms | HEENT^5^ | 465 (85.5) | 79 (14.5) | <0.0001 |
|  | Cardiorespiratory | 480 (88.9) | 60 (11.1) | <0.0001 |
|  | Gastrointestinal | 369 (82.6) | 78 (17.4) | 0.04 |
|  | Musculoskeletal | 267 (80.9) | 63 (19.1) | 0.491 |
|  | Neurologic | 450 (81.5) | 102 (18.5) | 0.452 |
|  | Dermatologic | 40 (87.0) | 6 (13.0) | 0.21 |
| Abnormal physical exam findings | Overall exam | 134 (88.2) | 18 (11.8) | 0.004 |
|  | HEENT | 89 (85.6) | 15 (14.4) | 0.133 |
|  | Cardiorespiratory | 28 (87.5) | 4 (12.5) | 0.37 |
|  | Gastrointestinal | 19 (100.0) | 0 (0) | 0.020 |
|  | Musculoskeletal | 9 (90.0) | 1 (10.0) | 0.70 |
|  | Neurologic | 78 (96.3) | 3 (3.7) | <0.0001 |
|  | Dermatologic | 45 (88.2) | 6 (11.8) | 0.175 |
| Malaria RDT^6^ result | Positive | 111 (67.3) | 54 (32.7) | 0.004 |
|  | Negative | 266 (79.2) | 70 (20.8) |  |
| Number of provisional diagnoses | 0^7^ | 11 (23.9) | 35 (76.1) | <0.0001 |
|  | 1 | 356 (80.0) | 89 (20.0) |  |
|  | 2 | 217 (90.4) | 23 (9.6) |  |
|  | 3 | 34 (79.1) | 9 (20.9) |  |
| Provisional diagnosis consistent with bacterial etiology^8^ | Yes | 273 (94.1) | 17 (5.9) | <0.0001 |
|  | No | 295 (77.4) | 86 (22.6) |  |

^1^ Chi-square test, fisher’s exact when expected cell count <5

^2^ Independent samples t-test

^3^ Quarter 1 (Q1) considered pre-COVID-19 pandemic, Quarter 2 (Q2) during COVID-19 pandemic

^4^ Rainy season considered March-May and November-December

^5^ Head, eyes, ears, nose, throat

^6^ Rapid diagnostic test

^7^ Includes both “unclear diagnosis at this time” and question left blank

^8^ Diagnoses considered possibly bacterial in etiology:  bacterial infection, ear infection, eye infection, gastroenteritis, meningitis, peptic ulcer disease, pneumonia, skin infection, tonsillitis/pharyngitis, tuberculosis, typhoid, lower respiratory tract infection, urinary tract infection
