# Supplementary material for: Antibiotic prescribing patterns at outpatient clinics in Western and Coastal Kenya
Source: PLOS Glob Public Health. 2025 Jan 3;5(1):e0004109. doi: 10.1371/journal.pgph.0004109 (PMC11698307; doi:10.1371/journal.pgph.0004109)
Supplement: S3 Table — 1 Chi-square test, fisher’s exact when expected cell count <5. 2 Independent samples t-test. 3 Quarter 1 (Q1) considered pre-COVID-19 pandemic, Quarter 2 (Q2) during COVID-19 pandemic. 4 Rainy season considered March-May and November-December. 5 Head, eyes, ears, nose, throat. 6 Rapid diagnostic test. 7 Includes both “unclear diagnosis at this time” and question left blank. 8 Diagnoses considered possibly bacterial in etiology: Bacterial infection, ear infection, eye infection, gastroenteritis, meningitis, peptic ulcer disease, pneumonia, skin infection, tonsillitis/pharyngitis, tuberculosis, typhoid, lower respiratory tract infection, urinary tract infection. (DOCX) [file pgph.0004109.s004.docx]

**S3 Table.** **Demographic and clinical characteristics of sick visits by antibiotic prescription for adults >18 years of age.**

| **Variable** | | **Antibiotic prescribed** | | **p-value^1^** |
| --- | --- | --- | --- | --- |
|  |  | **Yes, N (row %)**  **Total N= 456** | **No, N (row %)**  **Total N=243** |  |
| Site | West | 398 (69.8) | 172 (30.2) | <0.0001 |
|  | Coast | 58 (45.0) | 71 (55.0) |  |
| Sex | Female | 326 (68.9) | 147 (31.1) | 0.003 |
|  | Male | 130 (57.5) | 96 (42.5) |  |
| Pre vs during COVID-19 pandemic^3^ | Q1 2020 | 17 (27.0) | 46 (73.0) | <0.0001 |
|  | Q1 2021 | 110 (71.0) | 45 (29.0) |  |
| Season^4^ | Rainy season | 194 (70.5) | 81 (29.5) | 0.018 |
|  | Dry season | 262 (61.8) | 162 (38.2) |  |
| Visit | Initial visit within month | 438 (64.7) | 239 (35.3) | 0.11 |
|  | Second visit within month | 18 (81.8) | 4 (18.2) |  |
| Fever on exam | ≥38 C | 29 (64.4) | 16 (35.6) | 0.936 |
|  | <38 C | 411 (65.0) | 221 (35.0) |  |
| Duration of fever | ≥7 days | 431 (65.2) | 230 (34.8) | 0.77 |
|  | <7 days | 23 (67.6) | 11 (32.4) |  |
| Patient reported symptoms | HEENT^5^ | 334 (78.0) | 94 (22.0) | <0.0001 |
|  | Cardiorespiratory | 344 (77.0) | 103 (23.0) | <0.0001 |
|  | Gastrointestinal | 295 (71.8) | 116 (28.2) | <0.0001 |
|  | Musculoskeletal | 332 (64.8) | 180 (35.2) | 0.563 |
|  | Neurologic | 425 (66.5) | 214 (33.5) | 0.045 |
|  | Dermatologic | 24 (66.7) | 12 (33.3) | 0.869 |
| Abnormal physical exam findings | Overall exam | 72 (84.7) | 13 (15.3) | <0.0001 |
|  | HEENT | 35 (79.5) | 9 (20.5) | 0.04 |
|  | Cardiorespiratory | 12 (92.3) | 1 (7.7) | 0.041 |
|  | Gastrointestinal | 10 (71.4) | 4 (28.6) | 0.78 |
|  | Musculoskeletal | 11 (61.1) | 7 (38.9) | 0.89 |
|  | Neurologic | 101 (81.5) | 23 (18.5) | <0.0001 |
|  | Dermatologic | 18 (64.3) | 10 (35.7) | 0.91 |
| Malaria RDT^6^ result | Positive | 67 (58.8) | 47 (41.2) | 0.628 |
|  | Negative | 189 (61.4) | 119 (38.6) |  |
| Number of provisional diagnoses | 0^7^ | 20 (20.2) | 79 (79.8) | <0.0001 |
|  | 1 | 250 (67.4) | 121 (32.6) |  |
|  | 2 | 159 (80.3) | 39 (19.7) |  |
|  | 3 | 4 (14.3) | 24 (85.7) |  |
|  | 4 | 0 (0) | 3 (100.0) |  |
| Provisional diagnosis consistent with bacterial etiology^8^ | Yes | 225 (88.6) | 29 (11.4) | <.0001 |
|  | No | 158 (67.5) | 76 (32.5) |  |

^1^ Chi-square test, fisher’s exact when expected cell count <5

^2^ Independent samples t-test

^3^ Quarter 1 (Q1) considered pre-COVID-19 pandemic, Quarter 2 (Q2) during COVID-19 pandemic

^4^ Rainy season considered March-May and November-December

^5^ Head, eyes, ears, nose, throat

^6^ Rapid diagnostic test

^7^ Includes both “unclear diagnosis at this time” and question left blank

^8^ Diagnoses considered possibly bacterial in etiology:  bacterial infection, ear infection, eye infection, gastroenteritis, meningitis, peptic ulcer disease, pneumonia, skin infection, tonsillitis/pharyngitis, tuberculosis, typhoid, lower respiratory tract infection, urinary tract infection
